# Ultra-dense (~20 Tdot/in^2^) nanoparticle array from an ordered supramolecular dendrimer containing a metal precursor

**DOI:** 10.1038/s41598-019-40363-6

**Published:** 2019-03-07

**Authors:** Kiok Kwon, Bong Lim Suh, Kangho Park, Jihan Kim, Hee-Tae Jung

**Affiliations:** 1 National Research Laboratory for Organic Optoelectronic Materials, Department of Chemical and Biomolecular Engineering (BK-21 Plus), Korea Advanced Institute of Science and Technology (KAIST), Daejeon, 34141 Korea; 2 Department of Chemical and Biomolecular Engineering (BK-21 Plus), Korea Advanced Institute of Science and Technology (KAIST), Daejeon, 34141 Korea; 3 KAIST Institute for Nanocentury, Korea Advanced Institute of Science and Technology (KAIST), Daejeon, 34141 Korea

## Abstract

The fabrication of an ultra-dense, highly periodic nanoparticle array from a soft template is one of the most important issues in the fields of material science and nanotechnology. To date, block copolymer (BCP) structures have been primarily used as templates for fabricating highly periodic nanoparticle arrays with high areal densities. Herein, we demonstrate for the first time the use of a supramolecular dendrimer assembly for the formation of a highly ordered nanoparticle array with a high areal density of ~20 Tdot/in^2^, four times larger than that of the currently reported BCP-based nanoparticle arrays. By the simple thermal annealing of a dendrimers containing a metal precursor between two flat, solid substrates, a hexagonal array of small gold nanoparticles (with a diameter of ~1.6 nm and center-to-center distance of ~5.3 nm), oriented normal to the bottom, was achieved. Density functional theory calculations demonstrated that the gold cation strongly bound to the head group of the dendrimer. This structure served as a building block for self-assembly into a stable cylindrical structure. We anticipate that this study will lead to the creation of a large family of supramolecular dendrimers that can be utilized as soft templates for creating periodic, ultra-dense nanoparticle arrays.

## Introduction

Fabricating a monodisperse, ultrahigh-density nanoparticle array is crucial for various applications, including high-density data storage, memory devices, and the synthesis of secondary nanomaterials^1–6^. A large amount of studies on the generation of highly periodic two- or three-dimensional nanoparticle arrays using structured templates originating from soft building blocks have been conducted. These include the use of block copolymer (BCP) and liquid crystal (LC) defects. In general, nanoparticle arrays have been obtained by the loading of precursor^7–10^ or nanoparticles^11,12^ onto BCP micelles, followed by the encapsulation of the preformed nanoparticles^13–17^ into the phase-separated BCP domain and/or BCP interfaces, and finally precursor loading^3,18–20^ of or metal deposition^21,22^ of on the nanopatterned template of the BCP. In addition, ultra-dense, periodic nanostructure have been achieved by pattern transfer from ordered BCP domain from selective etching^23–25^. The defects of smectic liquid crystals^26–30^ and disclinations of cholesteric liquid crystals^31^ have also been utilized as templates for obtaining a periodic assembly of nanoparticles arrays. The areal densities of the nanoparticle arrays prepared from the aforementioned liquid crystal defects/dislocations or BCP templates range from 0.1 Gdot/in^2^ to 5 Tdot/in^2^.

## Results

### Computational calculation

In this paper, we describe a facile method for creating a highly periodic, ultra-high-density hexagonal array of small nanoparticles by using a supramolecular dendrimer as a new template to direct the self-assembly of nanoparticles. The nanoparticle array obtained by using this new method possess the highest areal density in comparison with the structured templates using existing soft building blocks, thus overcoming the limitations of the latter method. Our approach for achieving a highly ordered nanoparticle array is based on the self-assembly of supramolecular dendrimers containing a metal precursor (gold cations, Au^3+^).

**Figure 1:**
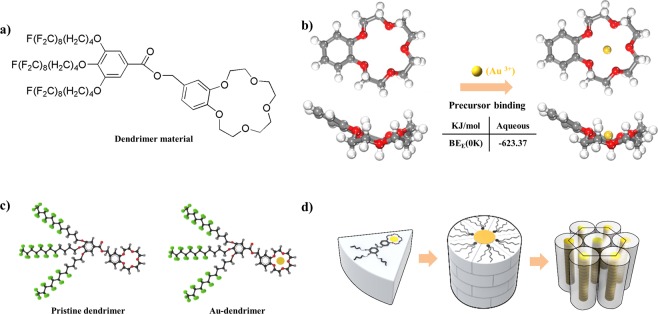
(**a**) Chemical structure of the supramolecular dendrimer. (**b**) Top and side views of the optimized structures of 15-*crown*-5 with attached benzene before (left) and after (right) gold cation (Au^3+^) binding in the gaseous phase. Au, C, O, and H are shown in gold, gray, red, and white, respectively. The binding energy of Au^3+^ on 15-*crown*-5 with an attached benzene (in kJ/mol) is included. (**c**) Molecular structure of the supramolecular dendrimer in the absence and presence of gold cation binding based on the DFT calculation. (**d**) Schematic illustration of the self-assembly of dendrimers containing gold cations (Au-dendrimers) into a cylindrical structure.

A dendrimer material (Fig. 1a) possessing a 15-*crown*-5 head, semi-fluorinated tails, and metal precursor (gold cations), was utilized as a building block to direct the formation of a highly ordered hexagonal array of gold nanoparticles within the thin film. The pristine supramolecular dendrimer material (Fig. 1b) is known to form a stable hexagonal cylindrical structure via a non-covalent self-assembly process^32,33^. For this technique, it is important to verify the detailed molecular structure of the dendrimer after metal precursor binding since the shape and chemistry of the basic building block is crucial to determining the ordered structure through the self-assembly.

To examine the change in the molecular structure of the dendrimer in the absence and presence of the precursor (Au^3+^) binding, we conducted density functional theory (DFT) calculation (Fig. 1b). Our DFT simulation result shows that the Au^3+^ cation is strongly bound in the cavity of the 15-*crown*-5 of dendrimer material with a high binding energy (Figs 1c and S1). This high binding energy may arise from the small ionic radius and high ionic charge of the gold cation (Au^3+^)^34–37^. Since the gold cation fits exactly into the cavity of the 15-*crown*-5, as shown in Fig. 1c, the dendrimer containing the gold cation (Au-dendrimer) has the same tapered shape and a similar molecular size as the pristine dendrimer (Fig. 1a,b). The Au-dendrimer is expected to form a stable hexagonal structure via spontaneous self-assembly, in which the core region of the supramolecular column is filled with gold cations (Fig. 1d). The highly periodic, ultra-dense gold nanoparticle hexagonal array can be achieved by the self-assembly of these Au-dendrimer building blocks. Herein, Au^3+^ gold ions was utilized a metal precursor for forming a complex with 15-crwon-5 moiety of dendrimer material since dendrimer-Au^3+^ complex showed the best result regarding a formation a highly ordered particle array compared to Au^1+^ ions. In addition, it is expected that Au^3+^ ion, having smaller ionic radius and large ionic charge compared to Au^1+^ ion, have the higher binding energy when they have complex with 15-crwon-5 moiety of dendrimer material based on the previous studies^36,38,39^.

### Mesophase behavior of Au-dendrimer

The fixation of the gold cation (Au^3+^) to 15-crown-5 of dendrimer material was achieved by mixing a gold salt solution (HAuCl_4_, 0.15 wt% MeOH) with a dendrimer solution (pristine dendrimer material, 0.2 wt% chloroform) at 2:1 volume ratio, with subsequent stirring for 4 h. The powder-type Au-dendrimer complex was obtained by solvent removal under vacuum for 1 h in the absence of light.

**Figure 2:**
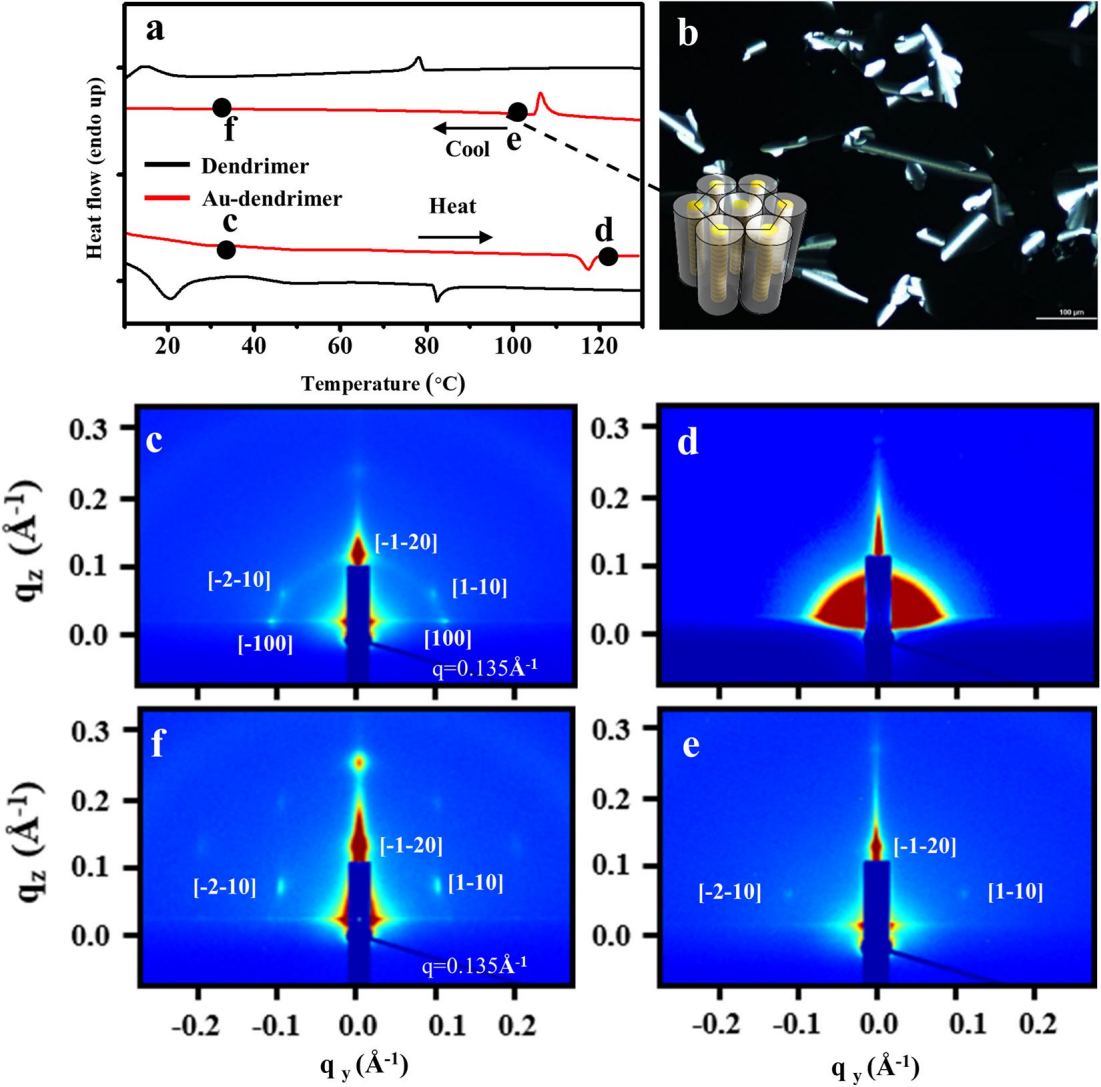
Phase behavior of the supramolecular dendrimers containing gold cations. (**a**) DSC of the first cooling and second heating run of the pristine dendrimers (dashed line) and Au-dendrimers (solid line). (**b**) POM image of the Au-dendrimers at 100 °C upon cooling from the isotropic melt. GISAXS images of the Au-dendrimer film obtained at 30 °C (**c**) and 120 °C (**d**) during the heating run, and at 115 °C (**e**) and 30 °C (**f**) during the cooling run.

To verify whether the pristine dendrimer maintained a cylindrical mesophase after precursor binding (as was expected), the phase behavior of the Au-dendrimer building block as a function of temperature was analyzed by differential scanning calorimetry (DSC, Fig. 2a), polarized optical microscopy (POM, Fig. 2b), and *in situ* grazing incident small-angle X-ray scattering (GISAXS, Fig. 2c–f). The pristine dendrimer undergo two phase transitions: crystalline-to-LC phase at 18 °C and isotropization at 78 °C (Fig. 2a, dashed line). Interestingly, the isotropic transition temperature changed from 78 °C to 118 °C (Fig. 2a, solid line) in the presence of the gold precursor; this may be attributed to the increased molecular weight and varied inter- and intramolecular interactions of the Au-dendrimer complex. To identify the mesophase that is formed upon cooling of this isotropic melt, we conducted POM analysis. A characteristic fan-shaped texture shown by POM imaging was obtained upon cooling of the Au-dendrimer complex from its isotropic state to room temperature. This is a typical texture indicating a columnar liquid crystalline phase (Fig. 2b). The phase behavior of the Au-dendrimer complex during heating and cooling was confirmed by *in situ* GISAXS in a single bottom–top open-air system (Fig. 2c–f). An approximately 100 nm thick Au-dendrimer film was formed by push coating^40^: a drop of Au-dendrimer solution (~1 μl, 0.5 wt% in chloroform) was placed onto a SiO_2_-coated amorphous fluoro-polymer (Teflon AF), covered with thermally cured PDMS (poly dimethyl siloxane) pad, and subsequently dried for 30 min at 25 °C in the absence of light. The scattering pattern of the as-casted film indicated the coexistence of vertical and planar orientations of the Au-dendrimer column to the bottom substrate (Fig. 2c). Three Bragg peaks at [1−10], [−1−20], and [−2−10] indicate the existence of a hexagonal array of Au-dendrimer supramolecular columns oriented parallel to the bottom substrate. In contrast, the two in-plane Bragg peaks at [100] and [−100] indicate the presence of supramolecular columns aligned vertically to the bottom substrate. The geometric confinement of the sandwiched cell by the PDMS top and bottom substrate resulted in the partial vertical orientation of supramolecular columns during the push coating process. The arc that is present around the scattering peaks originates from randomly oriented Au-dendrimer columns, the presence of which may be attributed to fast solvent removal during push coating. All diffraction patterns disappeared, indicating that the disordered state, above the isotropic transition temperature (118 °C, Fig. 2e). Three reflections ([1−10], [−1−20], and [−2−10]), began to emerge at q = 1.35 nm^−1^ as the temperature decreased below the isotropic transition temperature (Fig. 2e), which indicates the formation of a hexagonal array of Au-dendrimer columns. The average cylinder diameter was ~5.3 nm, which is slightly larger than that of a pristine dendrimer (ca. 4.5 nm). This increase in cylinder diameter might be due to the inclusion of the Au cation into the crown-ether core of the dendrimer, in which the varied chemistry of the Au-dendrimer complex can result in inter- and intramolecular interactions during the self-assembly process that are different to those in assembly of pristine dendrimer^32,41,42^. Decreasing to room temperature resulted in clearer scattering peaks with a second-order diffraction peak (Fig. 2f). This simple thermal treatment induced the formation of a highly ordered hexagonal array of Au-dendrimer columns within a short stabilization time (in the realm of minutes).

### Preparation of ultra-dense nanoparticle arrays

**Figure 3:**
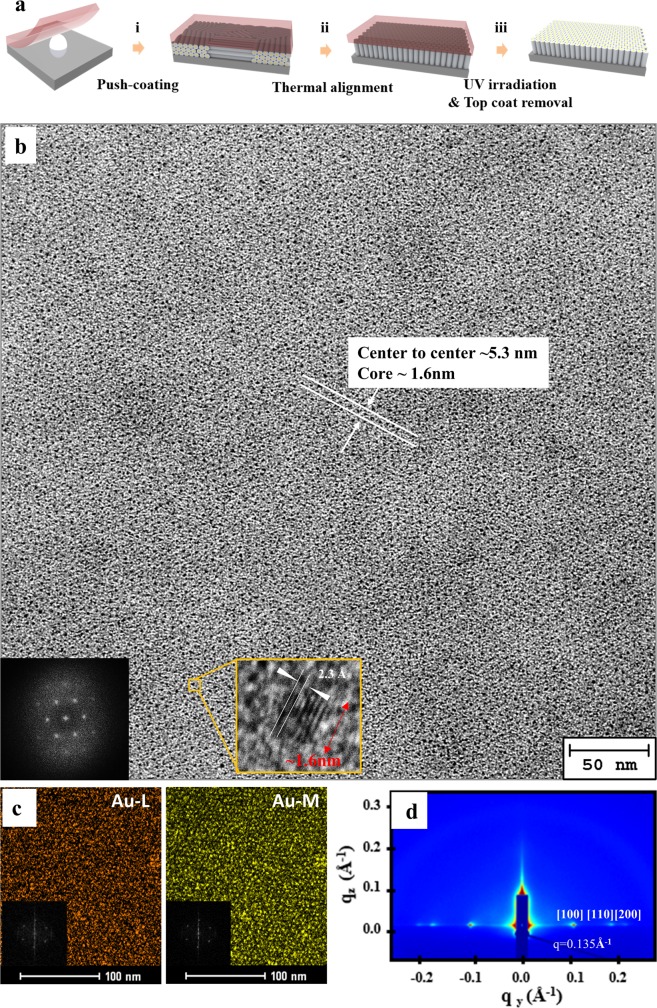
Fabrication of ultra-dense gold nanoparticle hexagonal array oriented normal to the bottom. (**a**) Schematic illustration of the deposition of the Au-dendrimer thin film on the bottom substrate by push coating, thermal annealing in a double-sandwich cell, UV irradiation, and removal of the top PDMS layer. (**b**) TEM images of a highly periodic hexagonal array of Au-dendrimers ([Au]/[Dend] = 1) oriented normal to the bottom substrate and without chemical staining, and the corresponding FFT pattern with a higher diffraction (bottom inset in Fig. 2a). Gold particles ~1.6 nm in size are clearly shown in the high-resolution TEM image with 2.3 Å d spacing (yellow inset in Fig. 2a). (**c**) Elemental mapping of Au (Au-L and Au-M) with six clearly shown spots in the FFT pattern (inset). (**d**) 2D GISAXS scattering patterns of the hexagonal cylinders oriented perpendicular to the bottom surface.

To prepare an ultra-dense periodic nanoparticle array, the orientation of the Au-dendrimer cylinders was controlled by using a double-sandwich cell as a geometric confinement template. Figure 3a shows the highly ordered hexagonal array of Au-dendrimer cylinders, oriented normal to the bottom, formed in the double-sandwich cell via simple thermal annealing. An approximately 100 nm thick Au-dendrimer film was formed by push coating: PDMS was used to cover a drop of Au-dendrimer solution (~1 μl, 0.5 wt% chloroform) that was placed onto an amorphous fluoro-polymer (Teflon AF)-coated bottom substrate (carbon-supported TEM grid). Subsequent drying was carried out for 30 min at 25 °C in the absence of light (i of Fig. 3a). The dried Au-dendrimer film, which was confined between the PDMS top and the bottom substrate, was heated above its isotropic temperature (118 °C) and cooled down to room temperature at a rate of 1 °C/min to induce a highly ordered hexagonal array of Au-dendrimers with a vertical orientation (ii of Fig. 3a). In this process, the top and bottom substrates serve as a geometric confinement template via the created double-sandwich system, which induces a highly ordered vertical alignment of the Au-dendrimer cylindrical structures^43–45^. An amorphous fluoro-polymer was coated on the bottom substrate to secure conformal contact between the Au-dendrimer film and the bottom substrate, which is crucial to the creation of a large-area vertical array of fluorinated, tapered dendrimers in the double-sandwich system^43^. After alignment was achieved, the Au-dendrimer film was exposed to UV (365 nm) for 1 h to obtain fully reduced gold particles (Au^0^) with a PDMS cover (iii of Fig. 3a). Finally, the PDMS top cover was peeled off to expose the Au-dendrimer film on the bottom substrate (iii of Fig. 3a), transmission electron microscopy (TEM) analysis was conducted without chemical staining. For the dendrimer film with a low amount of loaded gold cations, chemical staining with RuO_4_ was conducted to enhance the mass contrast and electron irradiation stability during TEM analysis.

A representative TEM image of Au-dendrimer building block in the sandwiched cell shows a perfect hexagonal array of cylindrical structures oriented normal to the bottom substrate (Fig. 3b). Analysis of the [1−10], [120], [−110], [−2−10], and [−1−20] reflections in the fast Fourier transform (FFT) diffraction pattern of the TEM image further reveals the formation of highly ordered hexagonal cylinders (inset of Fig. 3b). It is important to note that this TEM image of the Au-dendrimers was taken without chemical staining (with RuO_4_). Thus, the dark regions of the cores of the cylindrical structures clearly represents a gold-rich region, which arises from the high electron density of Au that is located in the core of the cylindrical structure obtained by self-assembly. Gold cation inclusion in the head of the dendrimer resulted in enhanced mesophase thermal stability and mass contrast, which made it possible to directly observe the ordered particle array without damaging the dendrimer film in the absence of chemical staining. A thorough inspection of the TEM image of the ordered structure generated from the Au-dendrimers revealed the existence of a highly periodic structure with an average cylinder diameter of ca. 5.3 nm, which is consistent with the GISAXS analysis shown in Fig. 2. A high-magnification TEM image of the Au-dendrimer column clearly shows the presence of Au nanoparticles (~1.6 nm) in the core region of the supramolecular cylindrical structure with 2.3 Å d spacing, which corresponds to the spacing between the [111] plane of crystalline Au (2.3 Å). The obtained gold nanoparticle array possesses the highest areal density (~20 Tdot/in^2^) in comparison with that of previously reported particle arrays prepared using other soft building blocks. Elemental mapping using energy-dispersive X-ray spectroscopy with a scanning transmission electron microscope was carried out to reconfirm the position of the gold nanoparticles on the Au-dendrimer film. Elemental mapping of Au (Fig. 3c, Au-L and Au-M) with six clearly visible spots in the FFT pattern reveals (inset of Fig. 3c, Au-L and Au-M) that the bright core region of the cylindrical structure in the dark-field scanning image (dark region of the bright-field TEM image) consists of gold species. This result confirms that the core region of the supramolecular columns, formed by the self-assembly of Au-dendrimers, becomes filled with gold species. Further evidence of the formation of vertically oriented Au-dendrimer films annealed in a double-sandwich cell was obtained by GISAXS. Clear in-plane Bragg peaks (Miller indices [100], [110], and [200] for a 2D hexagonal column) exist at q ≈ 1.35 nm^−1^ (period ca. 5.3 nm), $\sqrt{3}$ q ≈ 2.34 nm^−1^, and $\sqrt{4}$ q ≈ 2.7 nm^−1^, which suggest that the hexagonal columns are oriented perpendicular to the surface (Fig. 3d) over a large area.

### Reduction states of gold cation attached to dendrimer

**Figure 4:**
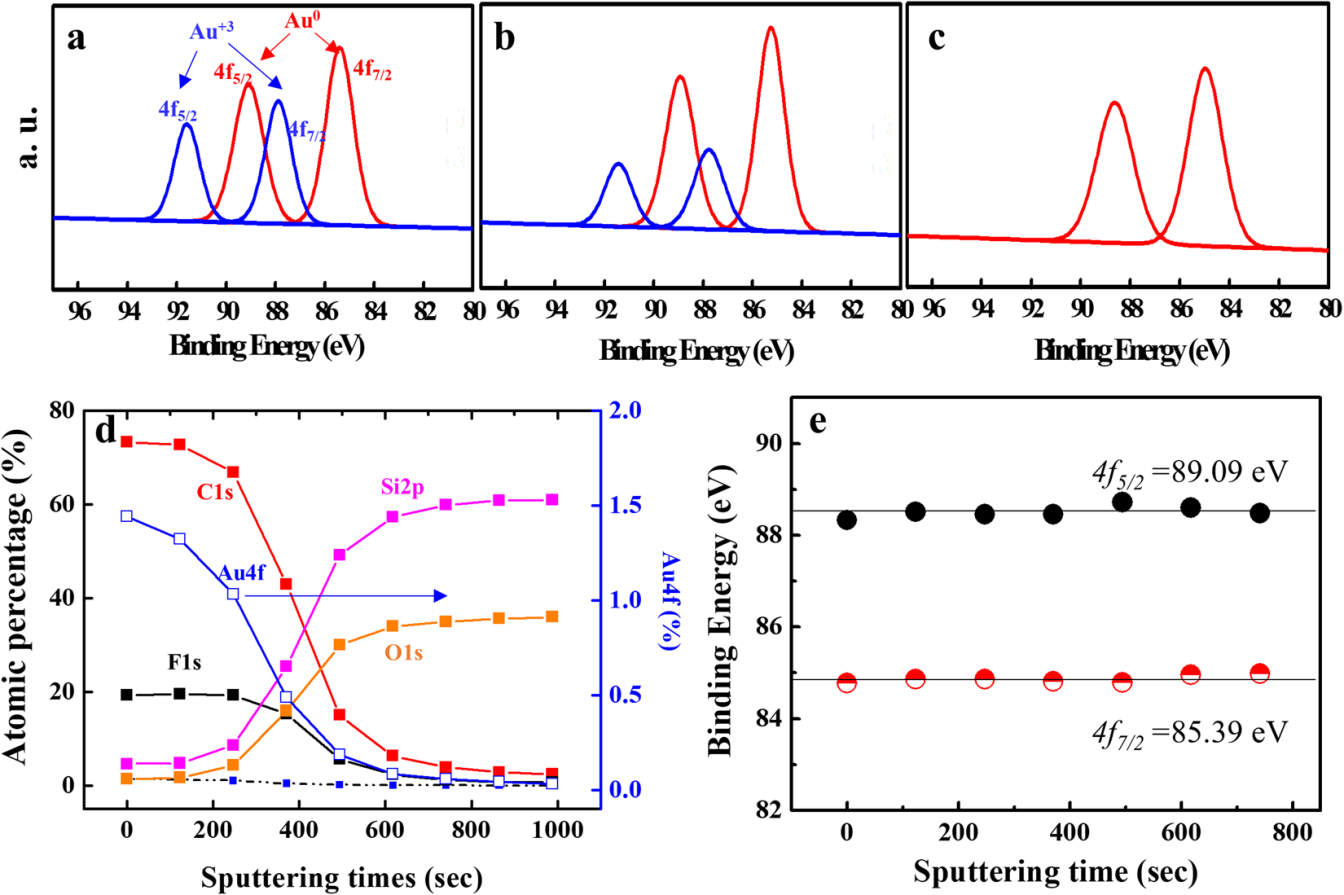
XPS Au4f region scans showing the difference in binding energy between Au^0^ and Au^3+^ of the (**a**) as-casted Au-dendrimer film, (**b**) thermally annealed Au-dendrimer film, and (**c**) fully reduced Au-dendrimer film via UV irradiation. (**d**) Relative atomic percentages of the elements gold, carbon, oxygen, fluorine, and silicon in the Au-dendrimer film as a function of the sputtering time. (**e**) Variation of the Au^0^ 4f binding energies of the fully reduced Au-dendrimer film (~100 nm thick) plotted from top to bottom as a function of the sputter time/depth.

X-ray photoelectron spectroscopy (XPS) measurement was conducted to trace the reduction state in each processing step: film formation, thermal annealing, and UV irradiation. As shown in Fig. 4a, XPS inspection of the as-casted Au-dendrimer film revealed an Au4f binding energy spectrum at 91. 58 eV (4f_5/2_ for Au^3+^), 89.09 eV (4f_5/2_ for Au^0^), 87. 88 eV (4f_7/2_ for Au^3+^), and 85.39 eV (4f_7/2_ for Au^0^), which indicate the coexistence of Au^3+^ and Au^0^ species in the as-casted Au-dendrimer films. This is attributed to the fact that a certain amount of Au^3+^ ions that are bound to the dendrimers were reduced to Au^0^ species during solution mixing and the push-coating process because of a reaction between the 15-*crown*-5 and gold cations^46,47^. In addition, a large amount of gold cations (Au^3+^) were reduced to Au^0^ species by the thermal annealing process (Fig. 4b) and subsequent UV irradiation (1 h at 365 nm), which resulted in formation of fully reduced gold nanoparticles (Fig. 4c).

To determine whether the gold nanoparticles were consistently present across the thickness of the film, an XPS depth-profiling analysis was carried out. Figure 4d shows the relative ratios of the elements gold, carbon, oxygen, fluorine, and silicon as a function of the sputtering time for the vertically oriented fully reduced Au-dendrimer film (Fig. 4d). The gold, carbon, and fluorine signals came from the Au-dendrimer films, while the silicon signals resulted primarily from the SiO_2_ wafer. The gold signals were present across the film thickness and decreased gradually until they disappeared toward the bottom of the films (at a sputtering time of 800 s; see Fig. 4d). In addition, the Au4f binding energy (Au^0^, 4f_5/2_ = 89.09 eV and 4f_7/2_ = 85.39 eV) obtained from seven points on the Au-dendrimer film (with a total thickness of ~100 nm and point-to-point thickness of ~16 nm) remained constant, confirming that fully reduced gold particles (Au^0^) exist throughout the thickness of the film (Fig. 4e). With the system used in this study, we can fabricate a highly ordered hexagonal array of small gold nanoparticles (~1.6 nm) throughout the thickness of the Au-dendrimer film with high periodicity. This was obtained by using supramolecular dendrimers containing a metal precursor as building block.

### Effect of [Au]/[Dend] on formation of nanoparticle arrays

**Figure 5:**
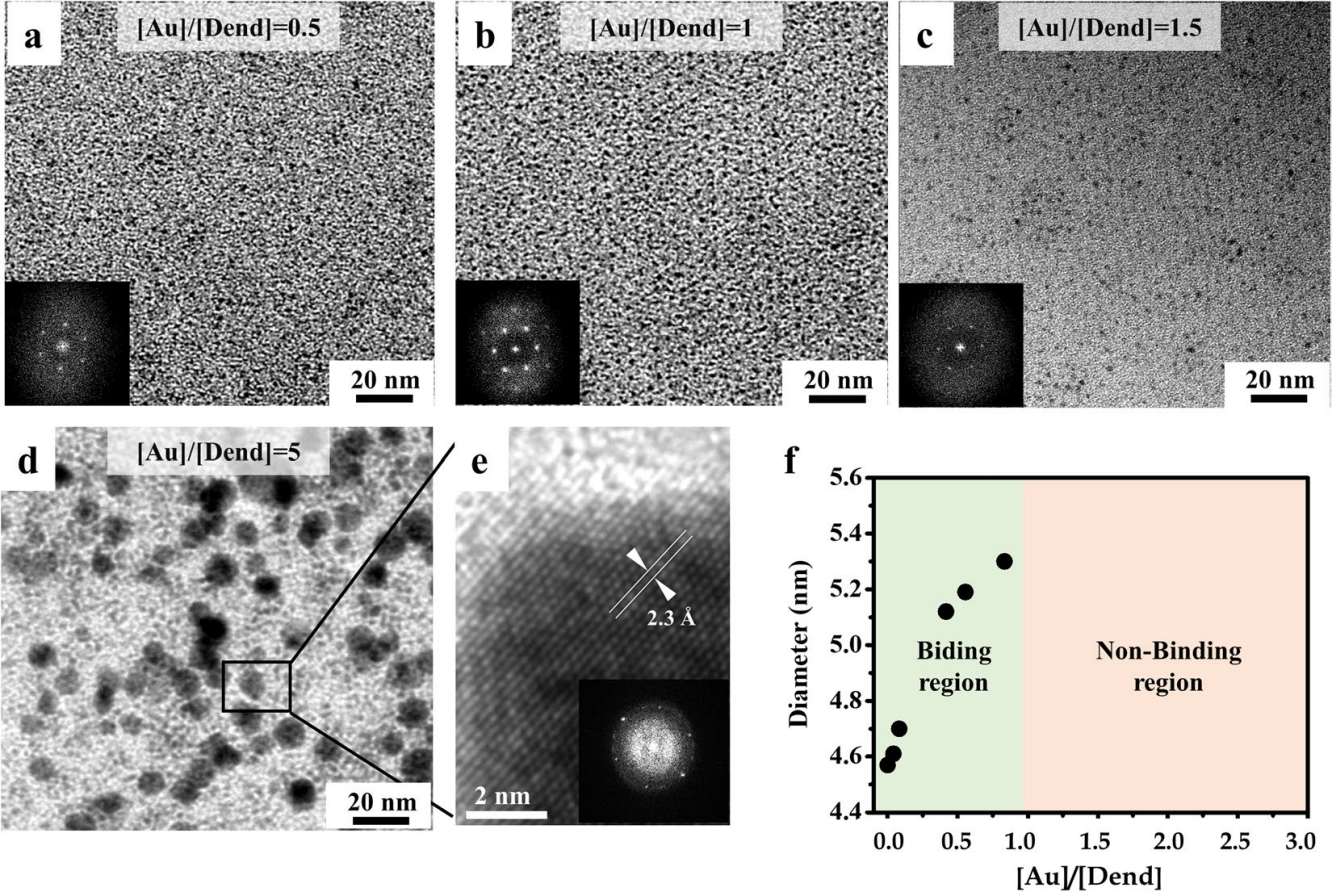
TEM images of the ordered structure of Au-dendrimers annealed between a Teflon AF coated bottom and PDMS top sandwiched cell at (**a**) [Au]/[Dend] ≈ 0.5, (**b**) [Au]/[Dend] ≈ 1, (**c**) [Au]/[Dend] ≈ 1.5, and (**d**) [Au]/[Dend] ≈ 5, and their corresponding FFT patterns (insets of **a**–**c**). (**e**) High-resolution TEM image of large gold nanoparticles formed at [Au]/[Dend] ≈ 5 and the corresponding FFT pattern (inset). (**f**) Variation of the diameter of the supramolecular cylindrical structures as a function of [Au]/[Dend].

The effects of the gold cations loaded onto the dendrimers on the ordered structure of the Au-dendrimer supramolecular assembly, was investigated by varying the molar ratios of the gold cations to dendrimers mixing solution. Herein, moles of gold cations and dendrimer molecules are denoted by [Au] and [Dend], respectively. The molar ratio of gold cations to dendrimer ([Au]/[Dend]) was varied from 0.04 to 5 (Figs 5 and S2). When [Au]/[Dend] was less than 1, a stable hexagonal cylindrical structure was successfully formed from the Au-dendrimer building blocks via thermal annealing in the double-sandwich cell. Even though we did not observe a clear TEM image at [Au]/[Dend] ≈ 0.5 because of a slight difference in mass contrast between the core and tail regions of the Au-dendrimer cylindrical structure, the six-spot FFT pattern (inset of Fig. 5a) that was observed confirmed the existence of a hexagonal cylindrical structure. The most ideal Au-dendrimer hexagonal array was obtained at [Au]/[Dend] ≈ 1 (Fig. 5b). As demonstrated in our DFT calculation, one gold cation fits into the cavity of a 15-*crown*-5 of dendrimer material, thus becoming an Au-dendrimer building block that can self-assemble into the desired hexagonal cylindrical structure. The dendrimer molecules bind the gold cations (Au^3+^) at this equivalent ratio of 1:1 to form the equivalent Au-dendrimer structure at [Au]/[Dend] ≈ 1. At [Au]/[Dend] ≈ 1.5; excess gold cations that do not bind to the dendrimers are likely to aggregate around the core region of the column, while the hexagonal array of the Au-dendrimer columns remains, as shown in Fig. 5c. When further gold cations were added to the dendrimer ([Au]/[Dend] ≈ 5, Fig. 5d), large gold particles (~10 nm in size, Fig. 5e) were generated, and the ordered hexagonal cylindrical structure was no longer found.

The average diameter of the supramolecular cylinders was calculated from TEM and GISAXS analysis (Table S1). Interestingly, the average diameter of the supramolecular cylinders in the range of 0≤ [Au]/[Dend] ≤1 gradually increased from 4.5 nm (pristine dendrimer, [Au]/[Dend] ≈ 0) to 5.3 nm (Au-dendrimer, [Au]/[Dend] ≈ 1) as the ratio of loaded gold cations to dendrimer (Fig. 5f, binding region) increased. Although the exact mechanism is not fully understood, it is likely that the pristine dendrimers and Au-dendrimers self-assemble together into a cylindrical structure, in which the varied molecular packing of supramolecular dendrimers leads to a gradual increase in cylinder diameter as a function of [Au]/[Dend]^32,41,42^.

### Preparation of other metal particle arrays

Our approach is not only limited to the use of gold cations (Au^3+^), as the pristine dendrimers can be used as templates for the formation of highly periodic arrays of various metal nanoparticles that possess an ionic radius and charge that allow them to bind into the cavity of the 15-*crown*-5 head of the dendrimer. Following the procedure described above, we conducted fixation experiments using this dendrimer and other cations such as silver (AgNO_3_) and platinum (PtCl_4_) at [cation]/[Dend] ≈ 1. Interestingly, highly periodic nanoparticle arrays of silver and platinum were generated via the self-assembly of the dendrimers containing metal cations in a double-sandwich cell composed of a Teflon AF bottom and PDMS top, as in the case of Au-dendrimers. The TEM images and corresponding FFT patterns shown in Fig. S3 clearly confirm the formation of hexagonal arrays of silver and platinum nanoparticles with average cylindrical diameters of 4.65 nm and 5.04 nm, respectively. These results demonstrate the possible use of these dendrimers as a new soft building block to guide a large amount of metal nanoparticles into forming hexagonal arrays with high periodicity and spatial accuracy in a simple manner. The spatial distribution of the nanoparticles might be controllable in the transition from a bilayer to rectangular columns, hexagonal columns, or cubic structures depending on the shape and chemical structure of the monodendron when the supramolecular dendrimer is used as a template to guide the nanoparticle assembly.

## Conclusions

In summary, we experimentally demonstrated, for the first time, the use of supramolecular dendrimers as a soft building block to guide a nanoparticle assembly. Highly periodic, ultra-dense nanoparticle arrays were generated by utilizing a dendrimer containing a metal precursor (Au-dendrimer), which formed a stable hexagonal cylindrical structure upon cooling from the isotropic melt. This investigation provides several significant insights for material science and nanotechnology. First, the nanoparticle arrays based on a soft building block formed in this study have the highest areal density (~20 Tdot/in^2^) with a ca. 1.6 nm dot size and ca. 5.3 nm interparticle spacing, as compared with those generated using existing soft building blocks. Second, this nanoparticle array is obtained in a simple and fast manner arising from beneficial properties of self-assembly promoted by the supramolecular dendrimers, such as the small features and fast stabilization of the ordered structure. Third, supramolecular dendrimers have a high possibility of being used as templates capable of forming a large family of metal nanoparticle arrays with a variety of orders. This is due to the hugely diverse chemistry, structure, and functionality of supramolecular dendrimers. We believe that this investigation presents the opportunity to create a large family of supramolecular dendrimers that are ultra-dense and possess a highly periodic nanopattern.

## Methods

### Material

Perfluorinated tapered supramolecular dendrimer material was synthesized by using the previously described method^43^. Fluorinated polymer (Teflon AF, Dupont) were used as received. PDMS pad (5 mm thick) was prepared by thermal curing of the pre-polymer (Silicone Elastomer base: curing agent = 10:1 weight ratio, Sylgard 184, Dow corning) on cleaned Si wafer at 80 °C for 2 h.

### Precursor fixation with dendrimer material

Fixation of gold cation (Au ^3+^) to the crown-ether group ([Au]/[Dend] =1) was conducted by mixing of gold salt solution (HAuCl_4_, 0.15 wt% in MeOH) with dendrimer solution (dendrimer material 1, 0.2 wt% in Chloroform) with 2:1 volume ratio, and subsequent stirring for four hours. Other Au-dendrimer complex having, different value of [Au]/[Dend], was obtained by varying the concentration of gold precursor in methanol proportional to value of [Au]/[Dend] with fixed concentration of dendrimer solution in chloroform (0.2 wt%). The powder type Au-dendrimer complex was obtained by solvent removal under vacuum for one hour with light blocked.

### Preparation of highly ordered hexagonal array of gold nanoparticles, oriented normal to bottom substrate, with the highest areal density

An approximately 100 nm thick Au-dendrimer film is formed by push coating, PDMS covering a drop of Au-dendrimer solution (a drop ~ 1 μl, 0.5 wt% in chloroform) on amorphous fluoro-polymer (Teflon AF) coated bottom substrate (carbon supported CF grid) and subsequent drying for 30 minutes at 25 °C with light blocked. The dried Au-dendrimer film, confined between PDMS top cover and bottom substrate, is heated above its isotropic temperature (118 °C) and cooled down to room temperature at 1 °C/min rate to induce a highly ordered hexagonal array of Au-dendrimer with vertical orientation. After the whole alignment process, Au-dendrimer film is exposed to UV (365 nm) for 1 hour to obtain fully reduced gold particle (Au^0^) with PDMS cover. PDMS top cover is peeled off. Transmission electron microscopy (TEM) analysis is conduct without chemical staining ([Au]/[Dend] >0.5). For the dendrimer film having low loaded gold cation ([Au]/[Dend] <0.5), chemical staining with RuO_4_ was conducted to enhance the mass contrast and electron irradiation stability during TEM analysis.

### *In-situ* GISAXS

GISAXS experiment was performed at Pohang Accelerator Laboratory (PAL), 3 C SAXS Line. An approximately 100 nm thick Au-dendrimer film is formed by push coating, PDMS covering a drop of Au-dendrimer solution (a drop ~ 1 μl, 0.5 wt% in chloroform) on amorphous fluoro-polymer (Teflon AF) coated SiO_2_ and subsequent drying for 30 minutes at 25 °C with light blocked. Ordering process is observed in real time during heating and cooling process in open air system.

### Computational details

The geometry of the 15-*crown*-5 head with an attached benzene group and the complex formed with Au^3+^ were optimized using the B3LYP functional^48^, which was implemented in the GAUSSIAN09 program (G09)^49^. The B3LYP density functional has been used to model the crown ether in other works^50,51^. The 6–31 + G(d) basis set was used to describe all of the electrons in the system. The Los Alamos effective core potential (LANL2DZ)^52^ was employed for the Au atom. The basis set superposition error was corrected for the binding energy value using the counterpoise method. To reduce the computational costs, the dendrimer material was simplified as a 15-*crown*-5 molecule with an attached benzene group. The expression for the DFT binding energy of a metal cation (Au^3+^) to the 15-*crown*-5 with an attached benzene group is defined as follows:

$$\begin{array}{c}{{\rm{BE}}}_{{\rm{E}}}={E(\mathrm{Au}}^{{\rm{3}}+} \mbox{-} \mathrm{15} \mbox{-} crown \mbox{-} 5\,{\rm{with}}\,{\rm{attached}}\,\mathrm{benzene})\\ \,\,\,-\{{\rm{E}}(\mathrm{15} \mbox{-} crown \mbox{-} 5\,{\rm{with}}\,{\rm{attached}}\,{\rm{benzene}})+{\rm{E}}({{\rm{Au}}}^{3+})\}\end{array}$$

where E(Au^3+^-15-*crown*-5 with attached benzene), E(15-*crown*-5 with attached benzene), and E(Au^3+^) are the total energies of the 15-*crown*-5 with attached benzene and Au^3+^ binding, the 15-*crown*-5 with attached benzene, and the Au^3+^ cation, respectively, all in the minimum energy configuration.

The binding energy of 15-*crown*-5 with an attached benzene complex in an aqueous phase was calculated at the same level by using the dielectric continuum solvation model. In modeling solvent effects in quantum chemical calculations, the polarizable continuum approach is widely used. The dielectric PCM (DPCM)^53^ is the original version of PCM that is no longer recommended^54^; as such, further variations of PCM (i.e., the conductor-like polarizable continuum model (CPCM)^55^ and integral equation formalism PCM (IEFPCM)^56^ are more commonly implemented in quantum chemistry packages. Among the available continuum solvent models (DPCM, CPCM, and IEFPCM), IEFPCM was used in this work; this is in keeping with a previous study indicating that IEFPCM provides accurate results for solvents with low dielectric constants^57^.

## Supplementary information

Supplementary material
