# Supplementary material for: Ultra-dense (~20 Tdot/in2) nanoparticle array from an ordered supramolecular dendrimer containing a metal precursor
Source: Sci Rep. 2019 Mar 7;9:3885. doi: 10.1038/s41598-019-40363-6 (PMC6405949; doi:10.1038/s41598-019-40363-6)
Supplement: Supplementary file 1 — Supplementary material [file 41598_2019_40363_MOESM1_ESM.pdf]

Supplementary material

Ultra-dense (~20 Tdot/in^2) nanoparticle array from an ordered supramolecular dendrimer containing a metal precursor

Kiok Kwon[1], Bong Lim Suh[2], Kangho Park[1], Jihan Kim[2], and Hee-Tae Jung [1,3*]*

[1] National Research Laboratory for Organic Optoelectronic Materials, Department of Chemical and Biomolecular Engineering (BK-21 Plus), Korea Advanced Institute of Science and Technology (KAIST), Daejeon 34141, Korea

[2] Department of Chemical and Biomolecular Engineering (BK-21 Plus), Korea Advanced Institute of Science and Technology (KAIST), Daejeon 34141, Korea

[3] KAIST Institute for Nanocentury, Korea Advanced Institute of Science and Technology (KAIST), Daejeon 34141, Korea

*Correspondence to: heetae@kaist.ac.kr, jihankim@kaist.ac.kr

Characterization

TEM: Direct visualization of particle array is conduct by using FE-TEM (200KV, Tecnai F20 at KARA) without chemical staining ([Au]/[Dend] > 0.5). For the dendrimer film having low loaded gold cation([Au]/[Dend] < 0.5), chemical staining with RuO_4 was conducted to enhance the mass contrast and electron irradiation stability during TEM analysis.

TEM_EDS: Elemental mappings were carried out using energy-dispersive X-ray spectroscopy in a scanning transmission electron microscope (STEM-EDS, 300KV, Tecnai G2 F30 S-Twin at KARA).

POM**:** Phase behavior of supramolecular dendrimer was investigated by polarized optical microscopy (POM, LV-100POL, Nikon) equipped with a hot stage and a charge-coupled device (CCD) camera. Characteristic POM image was obtained at room temperature under crossed polarized. Supramolecular dendrimer is filled into double sandwiched cell comprised

of two glasses with 2 μm spacing by capillary cation above its isotropic transition temperature, then, cooled down to room temperature.

XPS-analysis: The XPS measurement are carried out with a Thermo VG Scientific K-alpha, equipped with microfocused monochromator X-Ray source.

DSC: DSC measurement were performed in aluminum pans under nitrogen atmosphere using Netzsche DSC 204F at rate of 5°C /min.

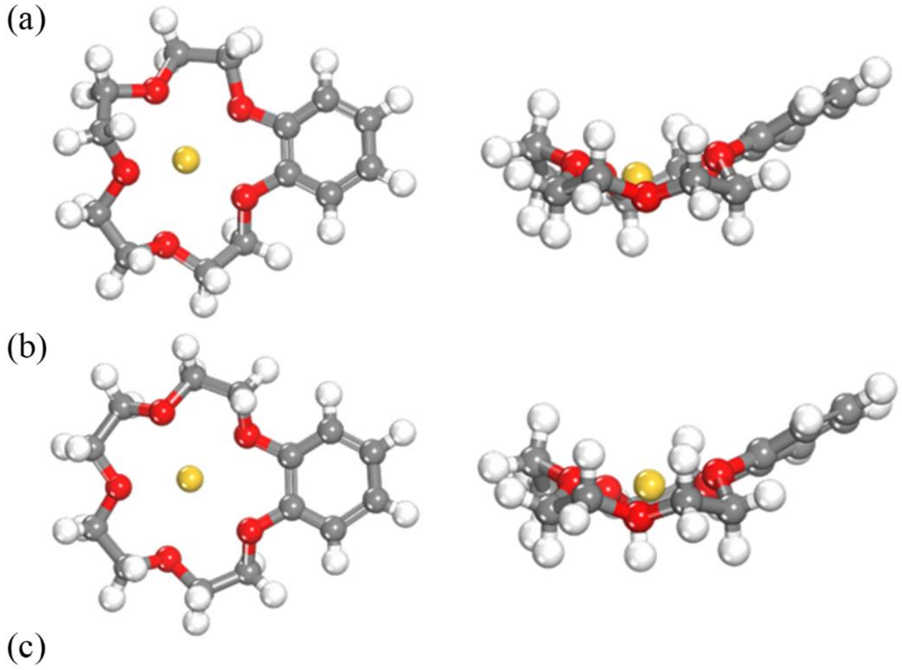

(c)

	Gas	Aqueous
$BE_E(0K)$	-2613.7	-623.37

Figure S1. (a) Top (left side) and side (right side) views of the optimized structures of Au^{3+}-15-Crown-5 with attached benzene. Au, C, O and H are shown in gold, grey, red and white colors, respectively. (b) Same as (a) but in the aqueous phase. (c) The binding energy of Au^{3+} on the 15-Crown-5 with attached benzene (in kJ/mol).

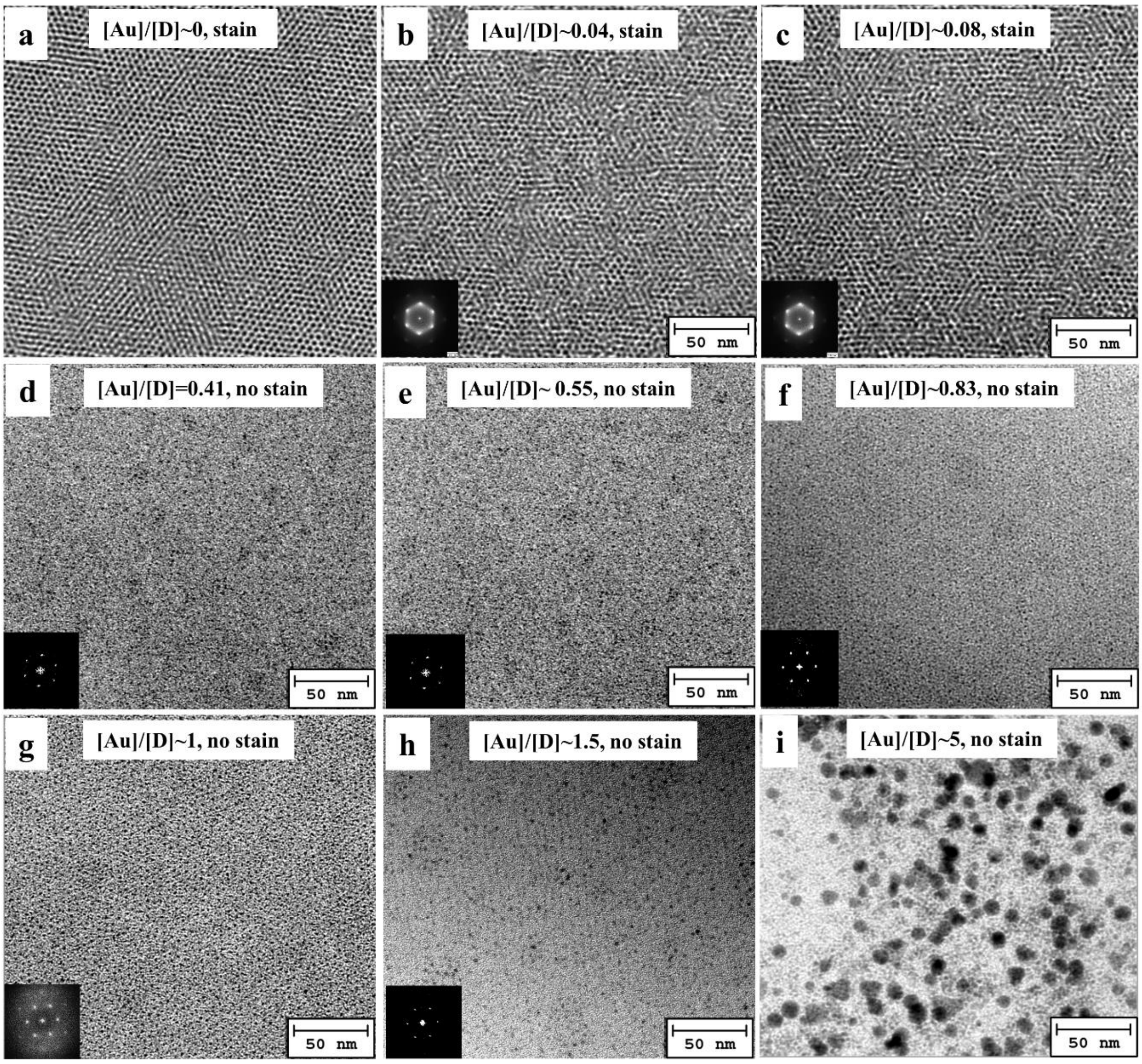

Figure S2. TEM image of ordered structure of Au-dendrimer annealed between Teflon AF coated bottom-PDMS top sandwiched cell at (a) [Au]/[Dend] ~ 0 and (b) ~ 0.04 with RuO_4 staining, (c) ~ 0.08 , (d) ~ 0.41, (e) ~ 0.55, (f) ~ 0.83, (g) ~ 1, (h) ~ 1.5 and (i) ~5 without RuO_4 staining.

[Au]/[Dend]	Ave. Diameter (nm)
0.00	4.57
0.04	4.61
0.08	4.70
0.41	5.12
0.55	5.19
1	5.30

Table S1. Average diameter of supramolecular cylindrical phase depending on the value of [Au]/[Dend], which was calculated from FFT patterns of TEM images of Fig.S2.

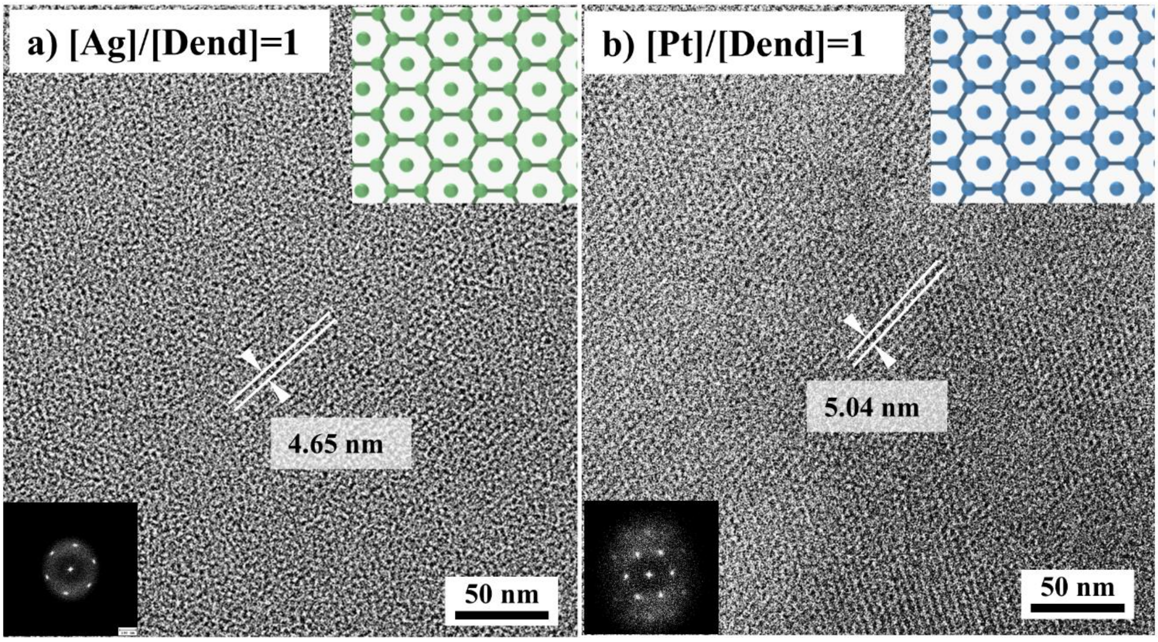

Figure S3. TEM image of ordered structure of (a) Au-dendrimer and (b) Pt-dendrimer, annealed between Teflon AF coated bottom-PDMS top sandwiched cell at [Cation]/[Dend] ~1 without RuO_4 chemical staining.
